# Gender Sensitive Depression in Male Patients with Alcohol and Substance Addiction: A Pilot Study

**DOI:** 10.1192/j.eurpsy.2025.1004

**Published:** 2025-08-26

**Authors:** I. G. Yilmaz-Karaman, T. Gündüz, B. Bağcı, E. Doğaneroğlu, G. Güleç

**Affiliations:** 1 Psychiatry, Eskişehir Osmangazi University, Eskişehir; 2 Psychiatry, İzmir Atatürk Training and Research Hospital; 3 Psychiatry, İzmir Katip Çelebi University Atatürk Training and Research Hospital, İzmir, Türkiye

## Abstract

**Introduction:**

Women are diagnosed with depression twice as often as men. Although depression is more common in women, men have higher suicide rates. Instead of seeking help, men tend to use various coping mechanisms that can be considered dysfunctional. These behaviors include risk-taking behaviors, aggressive behaviors, and alcohol and substance use. In addition, men’s failure to seek mental health services may be related to their beliefs about gender and masculinity. That symptom cluster is called a male depressive syndrome and can be seen in both sexes.

**Objectives:**

The present study aimed to measure depression with a widely used scale and a gender-sensitive scale among men with addiction disorders. Additionally, the study aimed to evaluate relations between addiction severity, depression and masculinity.

**Methods:**

Fifty-one male patients with alcohol and substance addiction participated in the present study. BAPİRT-alcohol and BAPİRT-drug questionnaires, Beck Depression Scale, Gender Sensitive Depression Scale, and Precarious Manhood Beliefs.

**Results:**

The mean age of the patients was 40.56 ± 12.35, and most were single (64.7%). Regarding the cut-off scores of BAPİRT scales, 45.9% of patients screened as high risk for substance addiction, and 86.27% screened as high risk for alcohol addiction. Depression scores were evaluated using the cut-off values. Both the Beck Depression Scale and the Gender Sensitive Depression Scale determined a depression prevalence of 60.8%, with diverse participants. Table 1 shows the intersection of the depression screening with the two scales.

**Table:** 

Depression scores	Beck Depression Scale (>16)		
	Positive	Negative	
Gender Sensitive Depression Scale (>49.5)	Positive	22 (43.1%)	9 (17.6%)
	Negative	9 (17.6%)	11 (21.6%)

**Conclusions:**

Male patients with addiction showed a high prevalence of depression (%60.8). Gender-sensitive depression was related to substance addiction severity.

Male patients with addiction may experience depression with a specific symptom cluster, which includes irritability.

**Disclosure of Interest:**

None Declared

